# The Semanticscience Integrated Ontology (SIO) for biomedical research and knowledge discovery

**DOI:** 10.1186/2041-1480-5-14

**Published:** 2014-03-06

**Authors:** Michel Dumontier, Christopher JO Baker, Joachim Baran, Alison Callahan, Leonid Chepelev, José Cruz-Toledo, Nicholas R Del Rio, Geraint Duck, Laura I Furlong, Nichealla Keath, Dana Klassen, Jamie P McCusker, Núria Queralt-Rosinach, Matthias Samwald, Natalia Villanueva-Rosales, Mark D Wilkinson, Robert Hoehndorf

**Affiliations:** 1 Center for Biomedical Informatics Research, Stanford University, Stanford, California USA; 2 Department of Computer Science and Applied Statistics, University of New Brunswick, Saint John, New Brunswick Canada; 3 Ontario Institute for Cancer Research, Toronto, Ontario Canada; 4 Department of Biology, Carleton University, Ottawa, Ontario Canada; 5 Cyber-ShARE Center of Excellence, University of Texas at El Paso, El Paso, Texas USA; 6 School of Computer Science, University of Manchester, Manchester, UK; 7 Hospital del Mar Medical Research Institute, Universitat Pompeu Fabra, Barcelona, Spain; 8 Digital Enterprise Research Institute, National University of Ireland, Galway, Ireland; 9 Department of Computer Science, Rensselaer Polytechnic Institute, Troy, New York USA; 10 Center for Medical Statistics, Informatics, and Intelligent Systems, Medical University of Vienna, Vienna, Austria; 11 Centro de Biotecnología y Genómica de Plantas, Universidad Politécnica de Madrid, Madrid, Spain; 12 Department of Physiology, Development and Neuroscience, University of Cambridge, Cambridge, UK

**Keywords:** Resource Description Framework, SPARQL Query, Relation Ontology, Open Biomedical Ontology, Resource Description Framework Data

## Abstract

The Semanticscience Integrated Ontology (SIO) is an ontology to facilitate biomedical knowledge discovery. SIO features a simple upper level comprised of essential types and relations for the rich description of arbitrary (real, hypothesized, virtual, fictional) objects, processes and their attributes. SIO specifies simple design patterns to describe and associate qualities, capabilities, functions, quantities, and informational entities including textual, geometrical, and mathematical entities, and provides specific extensions in the domains of chemistry, biology, biochemistry, and bioinformatics. SIO provides an ontological foundation for the Bio2RDF linked data for the life sciences project and is used for semantic integration and discovery for SADI-based semantic web services. SIO is freely available to all users under a creative commons by attribution license. See website for further information: http://sio.semanticscience.org.

**Electronic supplementary material:**

The online version of this article (doi:10.1186/2041-1480-5-14) contains supplementary material, which is available to authorized users.

## Background

Biomedical research is poised to enter an era of unprecedented large scale data analysis powered by hundreds of public biological databases and hundreds of millions of patient records. There is a real and urgent need to explore effective methods for biomedical data integration and knowledge management [1, 2]. Semantic-based technologies, such as ontologies, offer a proven method to exploit expert-based knowledge in the analysis of large datasets through terminological reasoning such as correspondence, classification, query answering and consistency checking [3–5].

The Semantic Web effort, as pursued under the auspices of the World Wide Web Consortium (W3C), provides a set of standards to facilitate the representation, publication, linking, querying and discovery of heterogeneous knowledge using web infrastructure [6]. In particular, the Resource Description Framework (RDF) [7] enables triple-based assertions about resources using web-friendly identifiers, RDF Schema (RDFS) [8] offers vocabulary to create terminological hierarchies, and the Web Ontology Language (OWL) [9] assists in the construction and interpretation of ontologies as sophisticated logic-based expressions to more precisely capture the meaning of types and relations between entities. With dozens of high value datasets now available in RDF and hundreds of biological ontologies expressed using OWL, there is a tantalizing opportunity to use these resources in knowledge discovery. Biomedical researchers have made use of Semantic Web technologies to uncover curation errors in systems biology models [10], find putative disease-causing genes [11], identify aberrant pathways [12], and uncover alternative drug therapies based on mechanism of action [13], among others [14]. These knowledge-based applications use automated reasoning over a coherent knowledge base often crafted from multiple and different underlying representations. Ontology-design patterns offer a simple way to guide users towards a uniform representation of knowledge [15–17].

With the goal of facilitating knowledge discovery through simple, but effective ontology-based data integration, we developed the Semanticscience Integrated Ontology (SIO). SIO offers classes and relations to describe and relate objects, processes and their attributes with specific extensions in the biomedical domain. Its relations cover aspects of spatial and temporal qualitative reasoning including location, containment, overlap, parthood and topology; participation and agency, linguistic and symbolic representation, as well as comparative and other information-oriented relations. Using straightforward mappings, we report on the substantial benefits afforded by SIO in the retrieval of RDF-based linked data and automatic composition of OWL-described semantic web services. Although SIO development is driven by needs in the biomedical domain, we show that SIO can be applied to a broader set of domains.

This paper is organized as follows: we first describe the current state of the SIO OWL implementation, and then we describe ontological foundations and essential relations in mereotopology, participation and reference. We then present three uses of SIO in knowledge representation and outline its use in the integration of data and web services. We finish with a brief comparison with related work. As a matter of convention, we use ‘single quotes’ to indicate labels, boldface to indicate **classes**, and italics to indicate *relations*.

### The semanticscience integrated ontology

**Figure 1:**
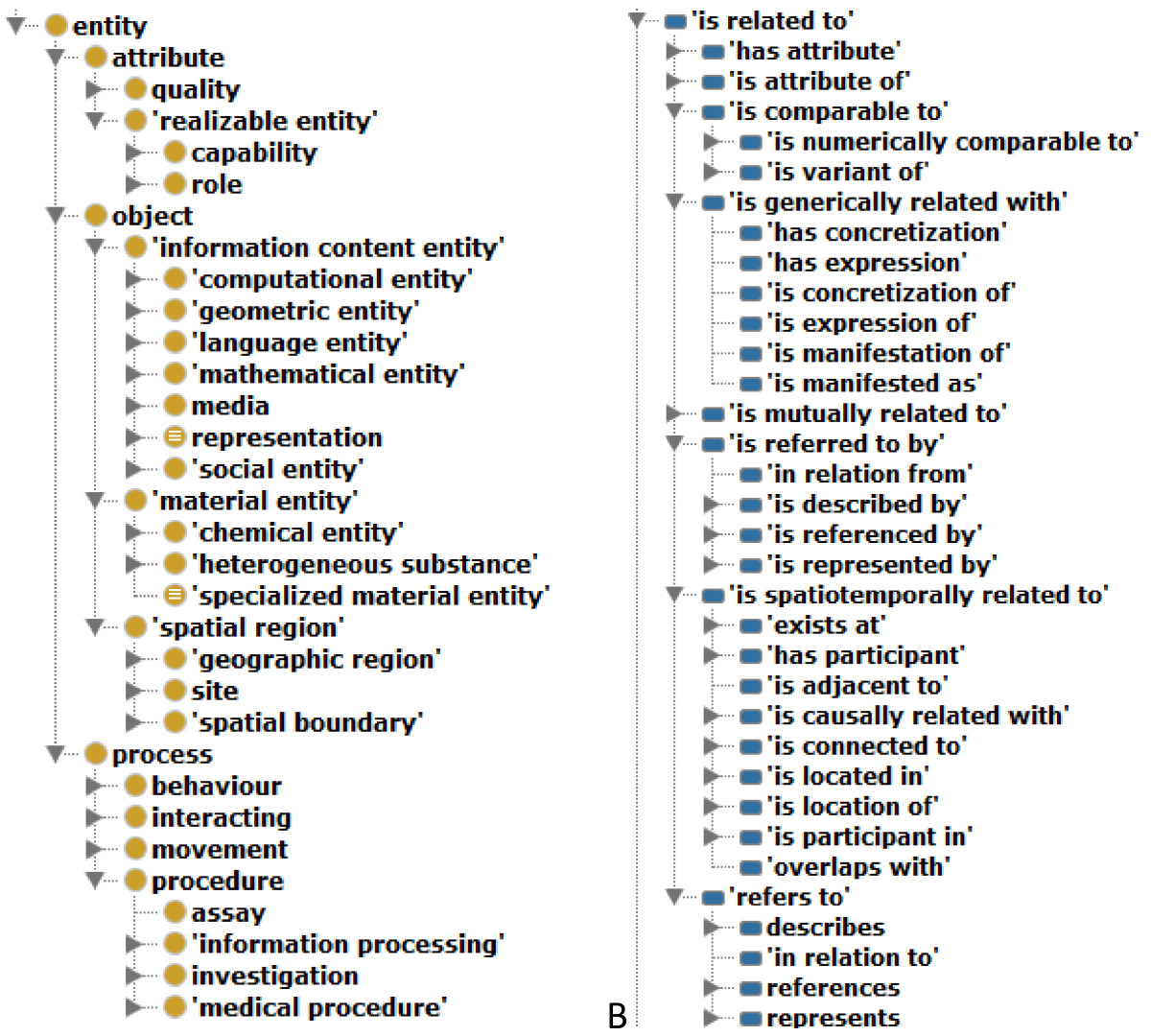
**Selected portions of (A) class and (B) object property hierarchies in SIO.**

As of November 2013, SIO (v1.0) is implemented as an OWL-DL ontology (SRIQ(D) expressivity) that comprises of 1396 classes, 203 object properties, 1 datatype property, 8 annotation properties, 7272 axioms, 1747 subClassOf axioms, 43 equivalentClass axioms, and 209 subPropertyOf axioms. English labels are provided using the *rdfs:label* annotation property while human readable, English language definitions are provided using the Dublin Core (dc:) Metadata term *dc:description*. The ontology has maximum depth of 41 subclasses while the average number of children is 2. Figure 1 shows a slice of the class and object-property hierarchies where **‘entity’** is the top level class and *‘is related to’* is the top level object property.

### Ontological foundation

**Figure 2:**
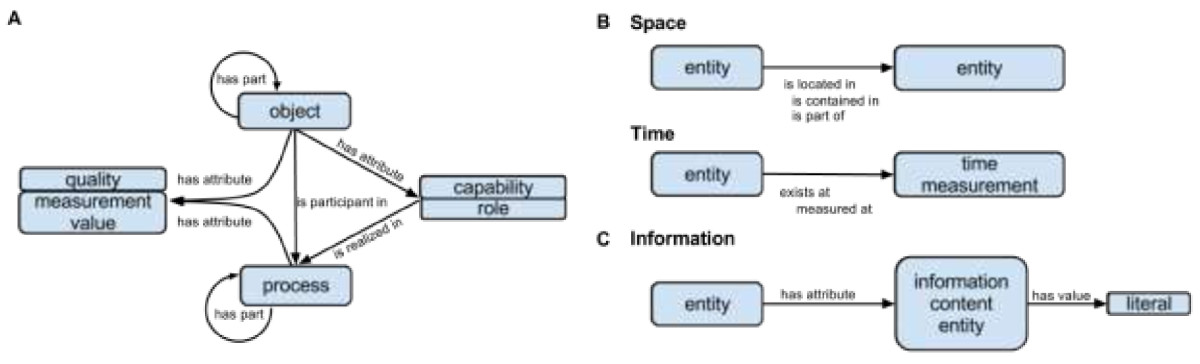
**Key objects and relations in SIO. (A)** Key SIO entities are objects, processes and their attributes (qualities, capabilities, roles, measurement values). Processes have objects as participants and may realize specific roles and capabilities. **(B)** Spatial and temporal qualification of SIO entities is captured through a set of relations (is located in, exists at) and sub-relations (e.g. is contained in, is part of, measured at), while **(C)** information in the form of literals (string, numbers, dates) are captured as instances of information content entities which are associated with their specific objects or processes.

SIO adheres to a three-dimensional worldview that is familiar to most scientists – one that distinguishes between processes and the objects that participate in them. An **‘object’** is an **‘entity’** that occupies space and is fully identifiable by its characteristics at any moment in time in which it exists. A **‘process’** is an **‘entity’** that unfolds in time and has temporal parts. While an entity ‘*exists at’* and ‘*is located in’* some space and time (Figure 2B), these need not be real space or real time, but may instead occur in a hypothetical (propositional), virtual (electronic), or fictional (creative work) setting. A **‘quality’** (intrinsic attribute), **‘capability’** (action specification) or **‘role’** (behavior, right and obligation) may exist at some time in the entity that bears it, but it *‘is realized in’* a process in which it plays a critical role (Figure 2A). The value of an **informational entity** such as a **‘measurement value’** (**‘quantity’** or **‘position’**) is represented as a literal - string, number (integer, float, double), boolean or date - using the ‘*has value’* data property (Figure 2C).

### Mereotopology

**Figure 3:**
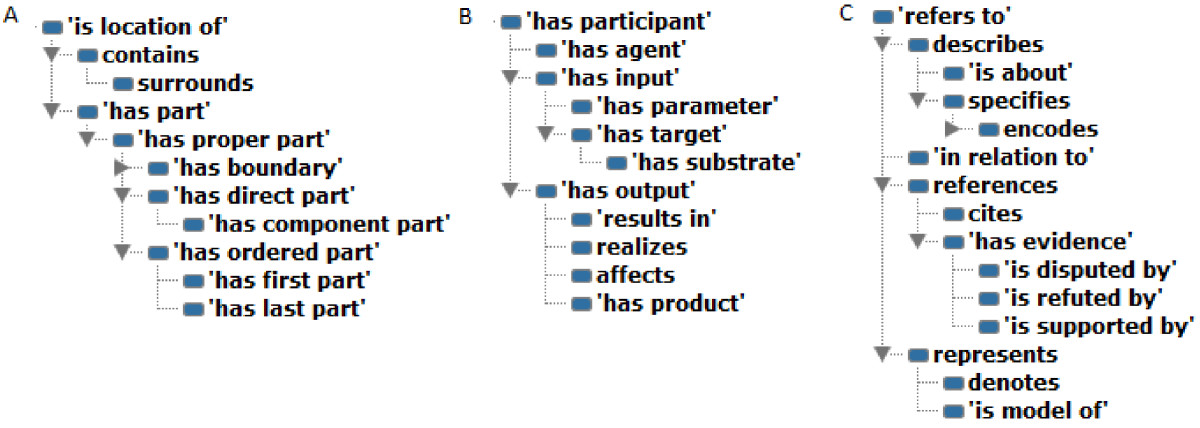
**Relation hierarchies for (A) mereotopological relations, (B) participatory relations, and (C) referential relations.**

SIO offers a number of mereotopological relations that can be used to describe one or more entities in terms of their spatial organization (Figure 3A). The parent relation *'is located of '* is a transitive relation that holds true if the spatial or temporal region of one entity fully overlaps with the spatial or temporal region of another entity. *'has part'* is a relation that is reflexive in the sense that the whole is a part of itself, and is also transitive in that a component of a part is also a component of the whole. Therefore, a query on the *‘has part’* relation will return the whole as an answer. *'has proper part'* is an irreflexive and asymmetric relation that ensures that the whole is different from and not one of its proper parts. '*has direct part'* enables users to quantify the number of parts (via a cardinality restriction) at a desired type granularity, which is not otherwise possible in OWL over the transitive ‘has part’ relation. *'has component part'* may be used to indicate that the part is intrinsic to the whole, and that the removal of the part changes the identity of the whole, with the caveat that there is no logic in OWL to directly infer this. *'contains'* is a transitive relation in which the 3D spatial region occupied by entity A fully overlaps with the spatial region occupied by entity B, but it is not the case that A has B as a part. *'surrounds'* is a relation that can be used to indicate that A *'contains'* B and either A *'is adjacent to'* B or A *'is directly connected to'* B.

**Figure 4:**
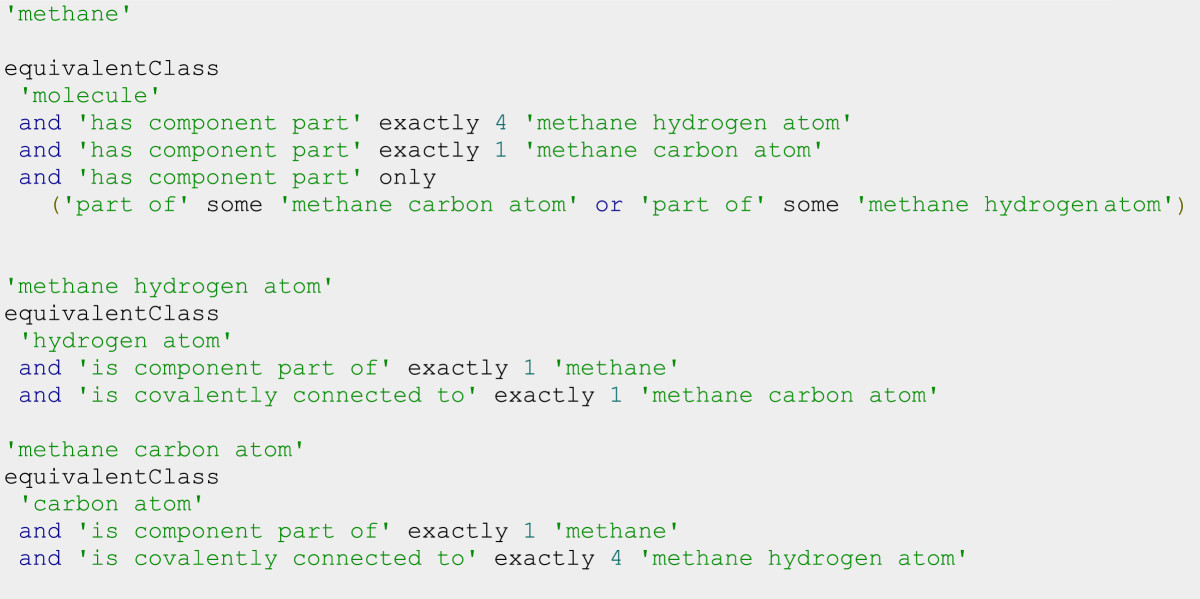
**Exact description of a molecule of methane using mereotopological relations in equivalent class axioms.**

The next set of mereotopological relations allows one to specify how the parts are positioned to one another. *'is connected to'* is a symmetric, transitive relation that specifies that components either directly share a boundary (they are directly connected to each other) or that they are indirectly connected by a path of unbroken direct connections. *'is directly connected to'* is a symmetric relation that indicates that two components share a boundary. Since this relation is non-transitive, we can use it in statements to quantify the number of connections from one part to other kinds of parts. *'is directly before'* is a relation between entities placed on a dimensional axis in which the projection of the position of the first entity is numerically less than the projection of the position of the second entity, and the entities are adjacent to one another. This is useful for indicating the spatial positioning of residues in linear biopolymers such as proteins or nucleic acids. A domain specific relation such as *‘is covalently connected to’* then enables one to describe the atomic connectivity within a molecule such as methane (Figure 4).

### Processes and participation

**Figure 5:**
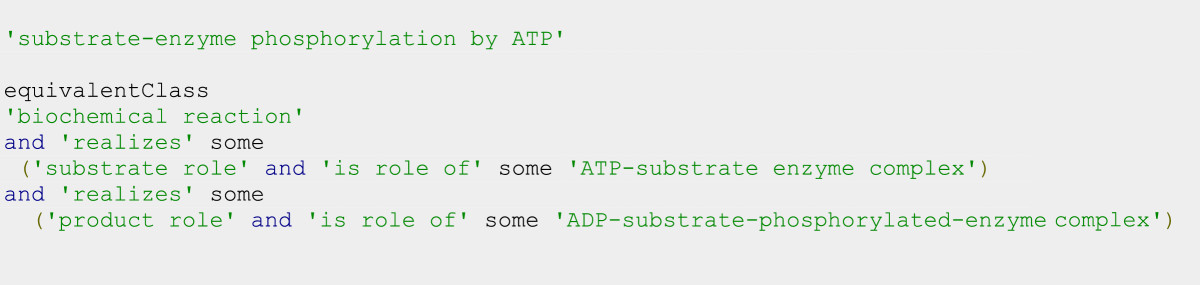
**Description of a process in terms of the participants and their roles.**

SIO provides a set of relations to describe processes in terms of their participants and their actions (Figure 3B). *'has participant'* indicates which entities participate in a process. *'has agent'* specifies entities that directly or actively participate in the process. *'has input'* specifies entities at the start of the process. *‘has parameter’* specifies those variables (and their values) used in the process. *'has target'* specifies entities that are modified during the process, but retain their identity. *'has substrate'* specifies entities that are consumed (or are sufficiently changed that they lose their canonical identity). *'has product'* specifies new entities formed as a result of a process. Relations such as *‘has substrate’, ‘has target’, ‘has product’* are examples of role-specialized relations. In SIO, more explicit role-based assertions can be formulated by stating that the role of an entity is realized in the process. For instance, Figure 5 shows a description of phosphorylation of an enzyme by ATP in which substrate and product roles are realized.

SIO includes an OWL2 property chain [realizes o is role of - > has participant] which enables an OWL2 DL reasoner to infer that entities having the realized role are also participants of the process.

### Referential relations

Referential relations in SIO are used to indicate what an object refers to or the nature of the mention of one entity by another (Figure 3C). At the top level, *‘refers to’* enables this basic mention, while *‘references’* is a relation where one entity mentions another, *‘describes’* is a relation where one entity provides a detailed account of another, and *‘represents’* is a relation where one entity is a sign, symbol or model for another. *‘describes’* is further partitioned into *‘is about’* where one entity provides information about another while *‘specifies’* contains specific information that can be used as evaluation criteria to determine the degree of conformance*. ‘references’* is further subdivided into *‘cites’* as a relation to refer to by way of example, authority or proof, and ‘*has evidence’* which is a relation between a proposition and something that demonstrates the truth of the assertion*. ‘has evidence’* has three sub-properties (*‘is supported by’, ‘is disputed by’, ‘is refuted by’*) which can articulate the type of evidence that one entity offers another. Finally, *‘represents’* is subdivided into *‘denotes’* which is a relation between an entity and what it is a sign or indication of, or what it specifically means, and *‘is model of’* which indicates that an artifact is a model or representation of another.

### Use cases

In this section we detail three use cases that outline how SIO can be used to represent biomedical knowledge, scientific experiments, and measurements.

### Biomedical knowledge

**Figure 6:**
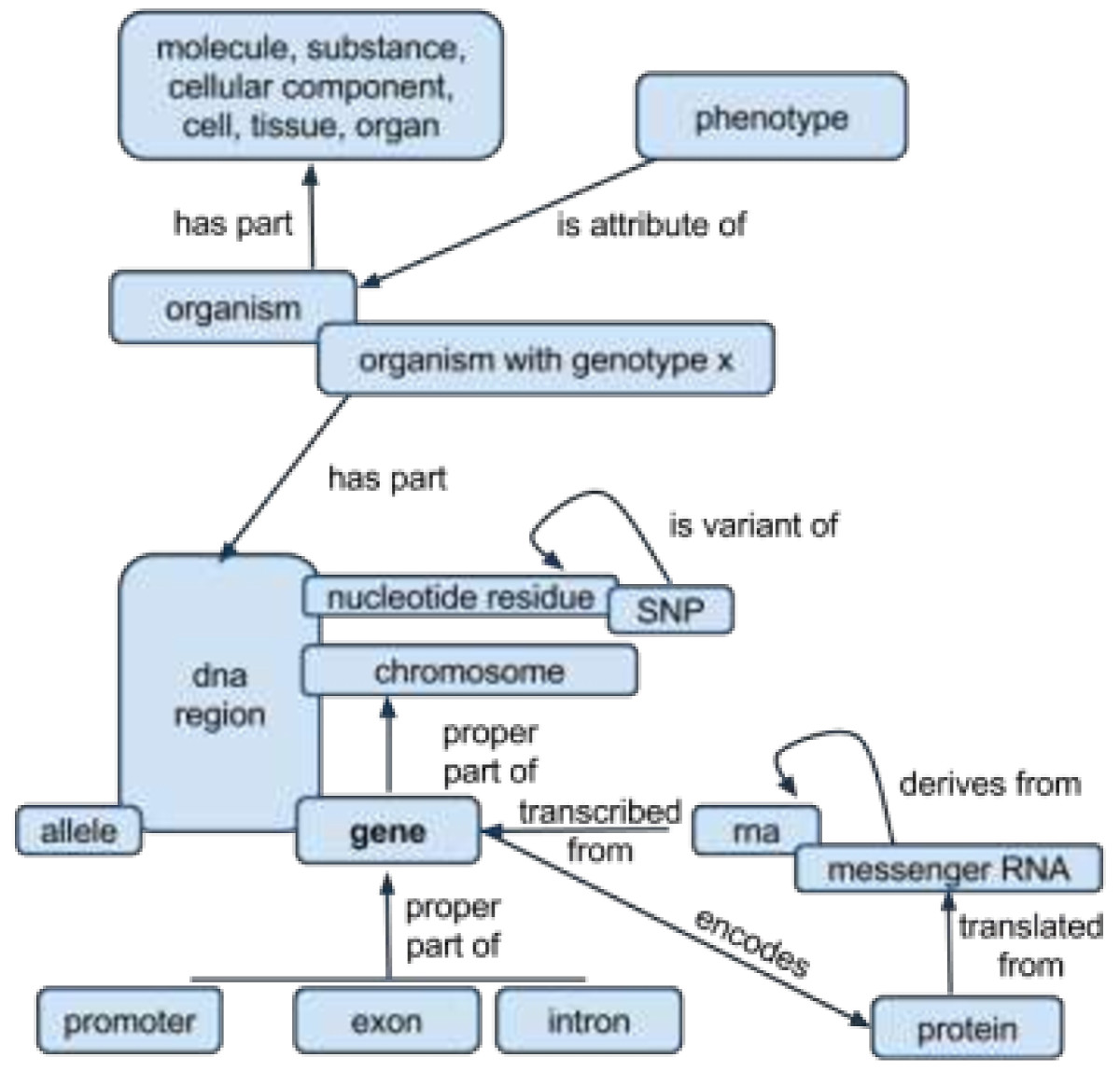
**Conceptual map of SIO entities and the relations between them as it pertains to molecular biology.** Rounded boxes indicate classes. Arrows indicate relations. Overlap of one concept on another indicates a subclass relationship (e.g. Gene is a type of DNA region).

In addition to the foundational classes and relations described above, SIO offers additional classes and relations to describe elements of biomedical interest including proteins, lipids, nucleic acids, small molecules, genotypes, phenotypes, biochemical reactions and pathways (Figure 6).

**Figure 7:**
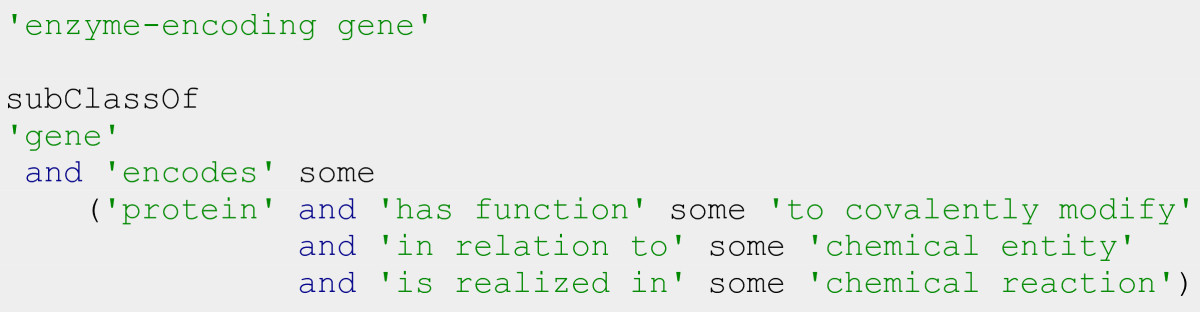
**Description of an enzyme-encoding gene.**

For instance, we can describe an enzyme-encoding gene as a gene that encodes a protein whose function is to covalently modify another chemical entity in some chemical reaction (Figure 7).

**Figure 8:**
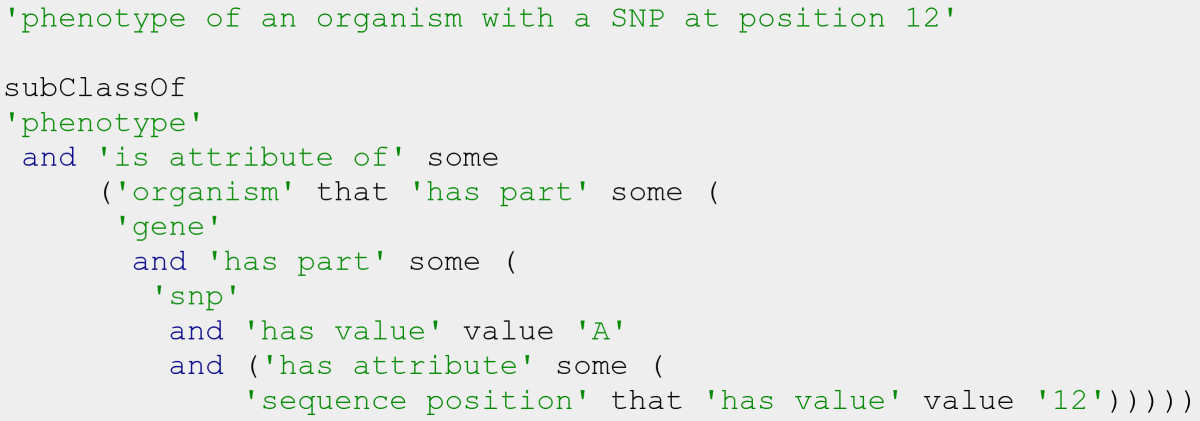
**Description of phenotype linked to a particular genotype.**

Phenotypes arising from specific genotypes can be expressed as attributes of an organism having a gene with a certain nucleotide (Figure 8).

### Scientific experiment

**Figure 9:**
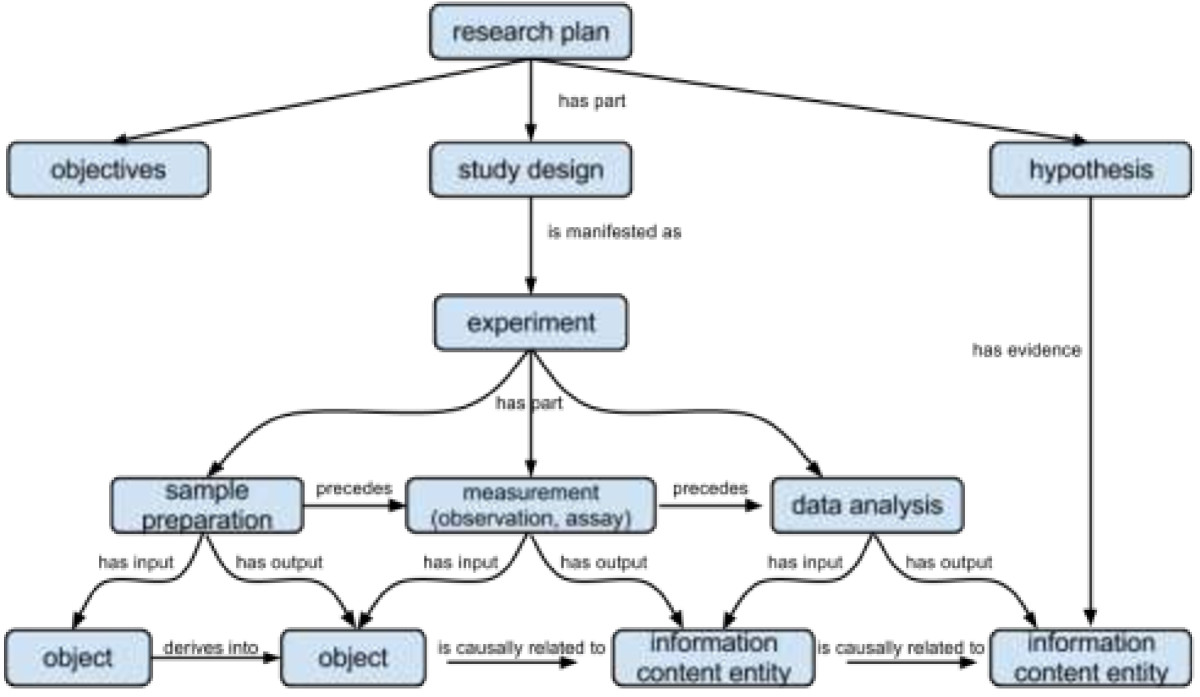
**Diagram illustrating major entities and their relations in a scientific experiment.**

In this use case, we describe the various parts and relationships within a scientific investigation. A scientific **‘experiment’** (Figure 9) is a **‘procedure’** that aims to support, dispute or refute a well formulated **‘hypothesis’** by **‘analysis’** of **‘data’** obtained through **‘observation’** and/or **‘measurement’**. Experiments *usually* involve:

- the development of a research **‘plan’** which includes, but is not limited to:
  - ○ the formulation of a **‘hypothesis’**
  - ○ the formulation of aims and **‘objectives’**
  - ○ the formulation of a **‘study design’**
- the execution of the research plan which includes, but is not limited to:
  - ○ the **‘selection, preparation or collection’** of a **‘sample’**
  - ○ the **‘collection of data’** through **‘observation’**, **‘assay’** or **‘measurement’**
  - ○ the **‘analysis’** of **‘data’**
  - ○ the preparation of an investigational **‘report’**

Figure 9 illustrates a pattern to express the relationship among a research plan, study design, experiment and its parts (*e.g.* sample preparation, measurement, analysis). Temporal parts are linked to the whole using SIO’s ‘*has proper part’* relation, while temporal ordering is achieved with SIO’s ‘*precedes’* relation.

A **‘description’** provides detailed information *‘about*‘ some **‘entity’** (**‘object’**, **‘process’** or **‘attribute’**), a **‘hypothesis’** is a proposed explanation of some phenomena, and an **‘objective’** is a description of a desired outcome. A description that *‘specifies*‘ a set of actions to be executed is an **‘action specification’** and include **‘plans’**, **‘study designs’**, recipes and **‘protocols’**. A plan should clearly identify (‘*specify*’) one or more **‘objectives’**, and optionally specify a **‘hypothesis’** or **‘study design’** as **‘attributes’**. A plan, like any action-based specification *‘is manifested as’* a **‘process’**. An objective ‘*is realized in*’ an experiment if and only if its outcomes are fully apparent. Data generated from the experiment may also serve as ‘*evidence for*’ the hypothesis, and more specifically found to be ‘*in support of’,* ‘*in disput* e *of*’, or ‘*in refutation of*’ the hypothesis. The Ontology for Biomedical Investigations (OBI) features more specific assays, material and data processing techniques [18].

### Measurements and measurement values

**Figure 10:**
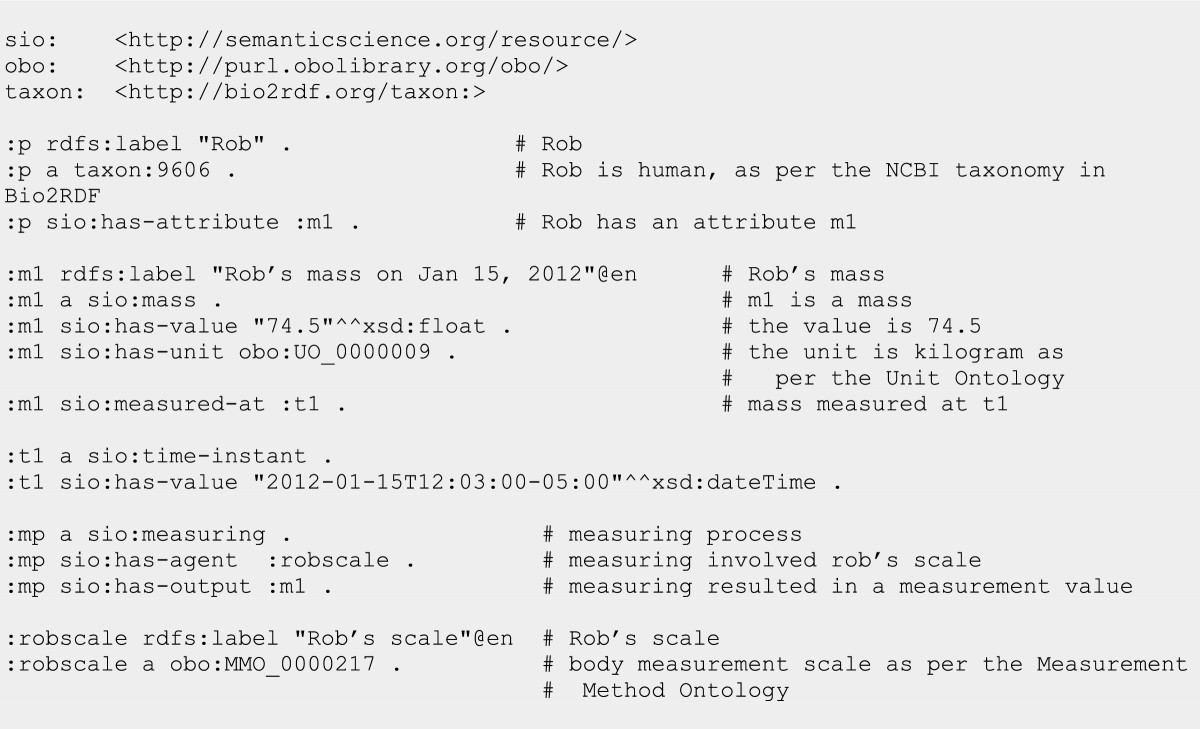
**Description of a mass measurement and value for an individual.**

Measurement values such as quantities or spatial positions are captured as information content entities (ICE), where the ICE is an attribute of the entity in question and the literal value is captured using SIO’s *‘has value’* data property. Units of measurements such as those defined by the Unit Ontology (UO) are indicated with SIO’s *‘has unit’* object property. The following RDF/N3 example (using the labels as URIs subset of SIO) shows how SIO captures Rob’s mass of 74.5 kg using a measurement scale on Jan 15, 2012 at 12:03 pm EDT (Figure 10).

### Applications

#### Semantic data integration and question answering

**Figure 11:**
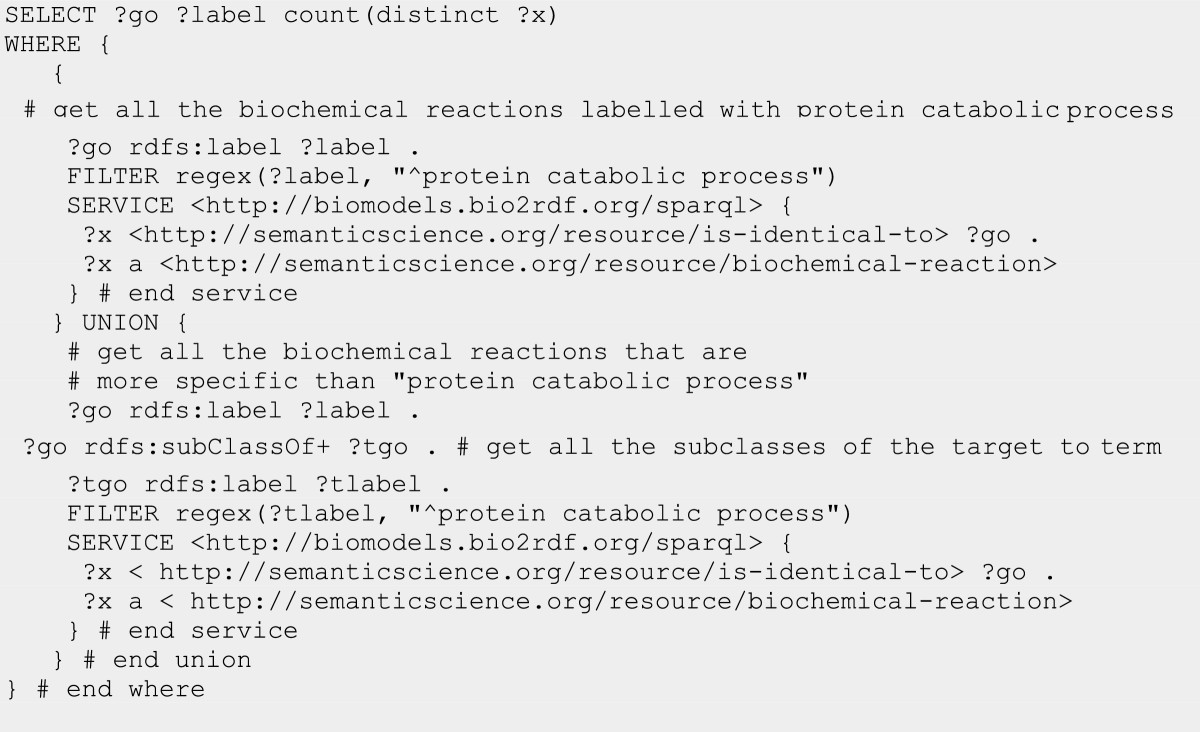
**SPARQL query that uses SIO to obtain biochemical reactions from the BioMODELS database where the reactions are annotated with the Gene Ontology term “protein catabolic process” or one of its subclasses.**

The Bio2RDF project uses Semantic Web technologies to offer the largest network of linked data for the life sciences [19]. Although the Bio2RDF approach provides minimal syntactic and referential interoperability (using RDF and a shared URI pattern), it does not address the issue of semantic interoperability across its datasets. Recent work [20] mapped SIO types and relations to Bio2RDF’s dataset-specific types and relations, thereby enabling SIO-based queries over linked data. In that work, resources such as DrugBank [21], the Pharmacogenomics Knowledgebase [22] and the FDA’s National Drug Code Directory (NDC) all provide drug information, and the types http://bio2rdf.org/drugbank_vocabulary:Drug, http://bio2rdf.org/pharmgkb_vocabulary:Drug, and http://bio2rdf.org/ndc_vocabulary:human-prescription-drug were mapped as subclasses of the SIO class **‘drug’**. Bio2RDF vocabulary mappings to SIO also make it possible to execute complex SPARQL queries over multiple Bio2RDF endpoints. For example, Figure 11 shows a SPARQL query that retrieves all the biochemical reactions in the Bio2RDF BioModels database [23] that are involved in the Gene Ontology (GO) term "protein catabolic process" or one of its subclasses.

This query is possible because the BioModels type for biochemical reaction has been mapped as a subclass of SIO’s **‘biochemical reaction’**. Similarly, the BioModels predicate for *‘is identical to’* has been mapped as a sub-property of SIO’s *‘is identical to’*.

### Semantic Web service interoperability

The Semantic Automated Discovery and Integration (SADI) framework consists of a set of design patterns for producing stateless Web Services that natively consume and produce RDF data [24, 25]. SADI services have been used to classify and annotate molecules based on their structure [26] and to uncover health information regarding drug-drug interactions [27]. The structure of the input and output data for SADI services are formally described by an input OWL class and an output OWL class respectively. Nearly 800 SADI services have been created as part of the C-BRASS (Canadian Bioinformatics Resources as Semantic Services) project. Consider for example, the abridged input and output descriptions for a SADI Web service [28] that executes BLASTN on the genome of *Prunus dulcis*. This service takes as an input a **‘deoxyribonucleic acid sequence’** and generates as output an instance of “BLASTedSequence that *‘has part’* some (**‘Subsequence’** that (*‘is part of’* some (**‘biopolymer sequence’**)) and *‘is part of’* some **‘BLASThit’**)”. Table 1 shows the top 10 SIO classes and relations that are directly referred to in the SADI global service registry.

**Table 1:** Top 10 classes and relations used in SADI services registered at http://sadiframework.org

Class	Frequency	Property	Frequency
Deoxyribonucleic acid sequence	159	Has part	289
Protein sequence	41	Is about	71
Ribonucleic acid sequence	21	Has attribute	67
Definition	2	Is attribute of	17
Name	2	Is part of	9
Sequence motif	2	Has output	9
Answer	1	Is derived from	9
Common name	1	Derives into	9
Description	1	Is similar to	9
Preferred name	1	Overlaps with	9

**Figure 12:**
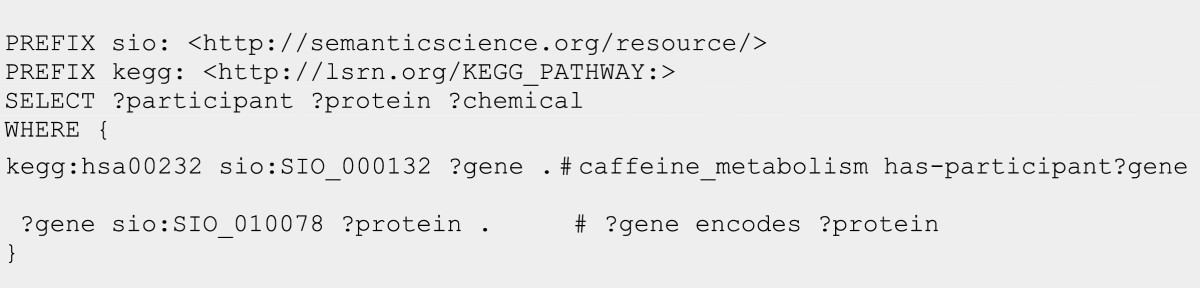
**SPARQL query to obtain proteins encoded by genes in KEGG pathways.**

SADI services can also be orchestrated into a computational workflow by matching the outputs of one service with the inputs of another service [25]. The SADI-aware SHARE client decomposes a SPARQL query into an executable workflow by matching the query components with SADI services. SADI uses an OWL reasoner to find appropriate matches between service inputs and outputs by finding those that subsume one another. In Figure 12, we show a SPARQL query that can be interpreted by the SHARE client to find proteins and compounds in the caffeine metabolism pathway (has00232). To answer this query, the SHARE client invokes three SADI services that wrap 2 existing BioMoby services that use SIO predicates *‘has participant’* and *‘encodes’*.

SIO is also being used in the Earth Life and Semantic Web (ELSEWeb) project to streamline the flow of heterogeneous geospatial data in order to ease the task of creating multi-source models of species-distribution [29]. ELSEWeb translates a family of industry standard XML geospatial metadata (e.g., OGC WCS, FGDC, CF) into RDF that is based on constructs defined by SIO and the Extensible Observation Ontology (OBOE) [30]. Geospatial satellite data is automatically discovered, transformed, and integrated with species distribution models services using the ELSEWebData ontology. The alignment of SIO, OBOE, ELSEWebData allows geospatial data to be queried and integrated with both data from the bio and environmental communities, providing a wider spectrum of modeling potential.

### Nanopublishing

**Figure 13:**
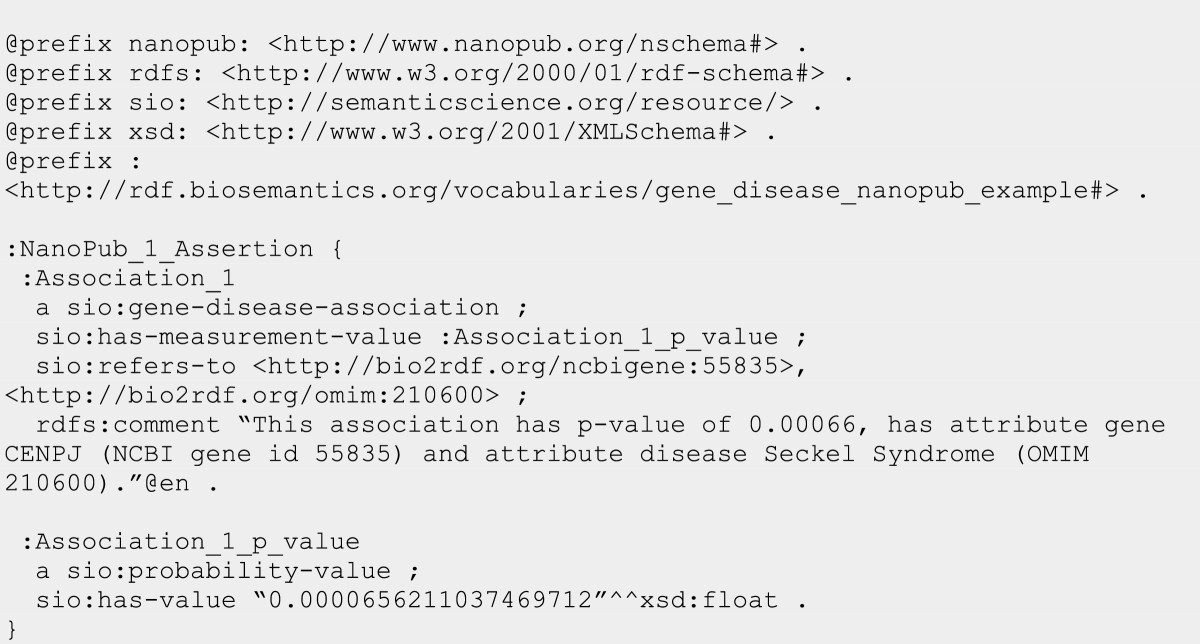
**The assertion portion of a nanopublication that uses SIO to express a text-mined association between the CENPJ gene and Seckel Syndrome, along with its probability-value.**

The publication of structured research data on the internet is an emerging area of interest. The nanopublication [31] is one effort that offers an RDF framework to capture assertions along with their provenance. Nanopublications have been used in enabling publication of genetic data [32] and more recently to arbitrary statements [33]. SIO is now being used to capture protein-protein interactions and gene-disease associations obtained through text mining [34]. To accommodate computed associations, we extended SIO with **‘association’** and more specific types including **‘gene-disease association’**. In Figure 13 we show a portion of a nanopublication that expresses an association between the gene CENPJ and Seckel Syndrome that was obtained through text mining. SIO is used to assert the type of association, the entities that are in the association (identified using Bio2RDF identifiers), and the associated p-value.

### Related work

The OBO Foundry is a collaborative effort to construct a set of orthogonal interoperable Open Biomedical Ontologies (OBO) [35]. OBO Foundry ontologies use the Basic Formal Ontology (BFO) as an upper level ontology for domain independent types and the Relation Ontology (RO) as a source of domain-independent relations. The BFO is a small (36 class) ontology that is intentionally limited by its realist philosophy to classes with at least one known instance and whose instances only exist in real space and time [36, 37]. In contrast, SIO simplifies the declaration and characterization of hypothetical, theorized or virtual entities (simply by virtue of having such a quality) and is thus more broadly applicable to situations of interest to the health care and life sciences including the presumed existence of underlying agents in medical disease or the existence of entities or attributes that are computationally predicted. SIO allows processes to have characterizing attributes, whereas the BFO does not [38]. The RO was initially [39] comprised of a collection of 8 domain-independent (*e.g.* has part) relations which has since been expanded to 160 relations, although these do not include all relations used in all OBO ontologies. OBO Foundry’s approach to building an interoperable set of ontologies can be contrasted with that of SIO, where instead of coordinating needs and duplication across dozens of ontologies, SIO serves as a single point of interoperability capable of addressing needs that go beyond its current scope. In order to foster semantic interoperability between SIO and BFO + RO, we have mapped 9 BFO classes and 24 RO relations to SIO (mapping available at [40]).

BioTop [41] is an upper level ontology for biology and medicine that features 390 classes and 82 object properties. The class top-level is characterized by a flattened set of basic categories (material object, immaterial object, information object, process, quality, role, condition, disposition, time, value region) while the object hierarchy provides type-specific relations around physical, processual and abstract nature (*e.g.* has physical part, has processual part, has abstract part). BioTop includes relatively sophisticated formalization for selected terms, for example pathological disposition is defined as "disposition that ('inheres in' some ('bearer of' some (canonicity and ('quality located' some 'noncanonical value region'))))", where SIO would simply express it as a 'biological disposition' that ('is attribute of' some ('entity' that 'has attribute' some 'pathological quality')). BioTop has been used to provide a number of ontology design patterns [42, 43] and to identify semantic type errors in the UMLS network [44].

The Translational Medicine Ontology (TMO) is a unifying ontology for chemical, genomic and proteomic data with disease, treatment, and electronic health records [13]. The TMO acted as a central schema that mapped basic types to dozens of bio-ontologies and linked open data. The utility of the TMO was demonstrated by answering a series of questions pertaining to diagnosis, prescription, drug mechanism of action, alternative therapeutics, and biomarkers. As SIO emerged from considerations in the TMO effort, SIO can be seen as the supported successor to TMO.

## Conclusion

The Semanticscience Integrated Ontology (SIO) is an ontology of basic types and relations to capture a wide span of knowledge through a set of emerging domain-specific patterns using RDF/OWL. SIO has emerged to support the demands of the bioinformatics community, with a special emphasis on biological knowledge representation as well as ontology, data and service interoperability.

### Availability

The SIO homepage is http://sio.semanticscience.org. SIO is freely available under a Creative Commons by Attribution license at http://semanticscience.org/ontology/sio.owl. Version 1.0 of SIO is available as Additional file 1. The base namespace for SIO entities (classes, properties) is http://semanticscience.org/resource/. SIO entities are identified using resolvable HTTP URIs, initially formulated as an alphanumeric identifier e.g. http://semanticscience.org/resource/SIO_000001, but is alternatively accessible using a label-based identifier e.g. http://semanticscience.org/resource/is-related-to. These and other generated subsets are available from http://goo.gl/0LgN8.

## Electronic supplementary material

Additional file 1:The Semanticscience Integrated Ontology, v1.0. This file in the format of OWL2, the Web Ontology Language. (ZIP 98 KB)

## Authors’ original submitted files for images

Below are the links to the authors’ original submitted files for images. Authors’ original file for figure 1 Authors’ original file for figure 2 Authors’ original file for figure 3 Authors’ original file for figure 4 Authors’ original file for figure 5 Authors’ original file for figure 6 Authors’ original file for figure 7 Authors’ original file for figure 8 Authors’ original file for figure 9 Authors’ original file for figure 10 Authors’ original file for figure 11 Authors’ original file for figure 12 Authors’ original file for figure 13
