# Supplementary figures and images for: The Semanticscience Integrated Ontology (SIO) for biomedical research and knowledge discovery
Source: J Biomed Semantics. 2014 Mar 6;5:14. doi: 10.1186/2041-1480-5-14 (PMC4015691; doi:10.1186/2041-1480-5-14)

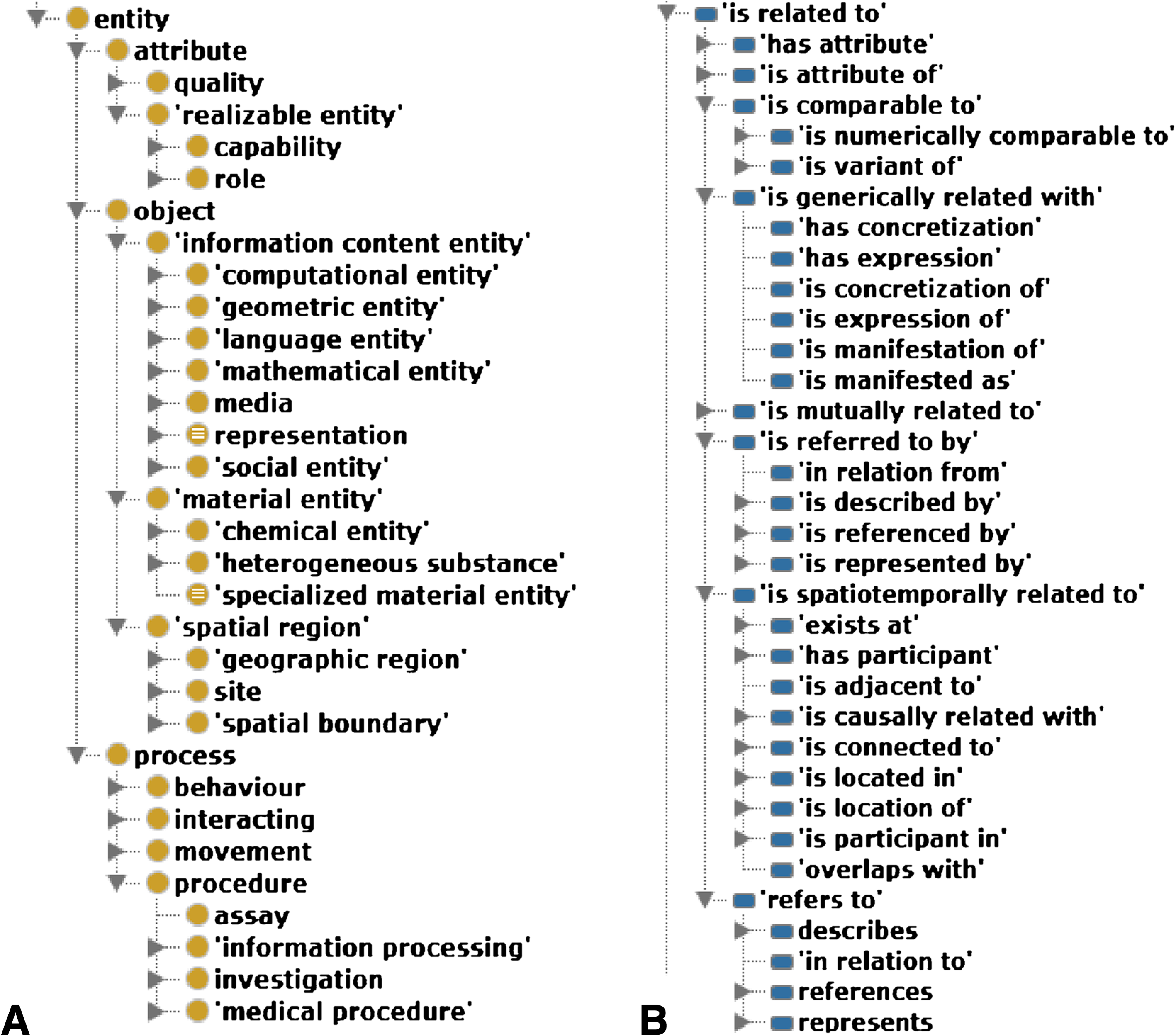

Supplement: Supplementary file 2 — Authors’ original file for figure 1 [file 13326_2013_202_MOESM2_ESM.tiff]

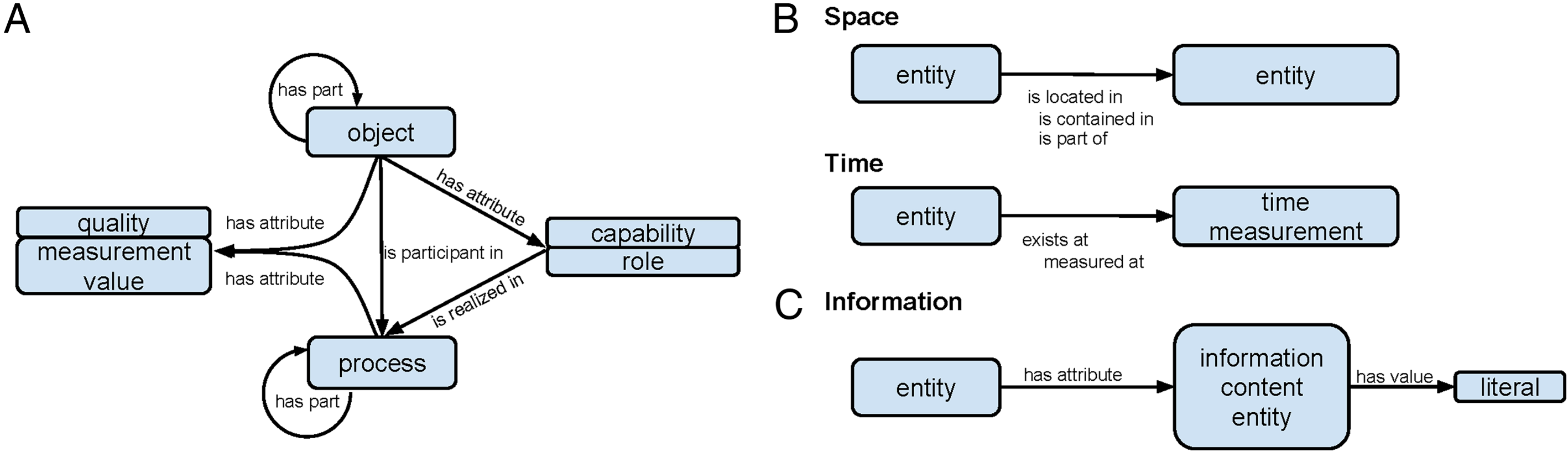

Supplement: Supplementary file 3 — Authors’ original file for figure 2 [file 13326_2013_202_MOESM3_ESM.tiff]

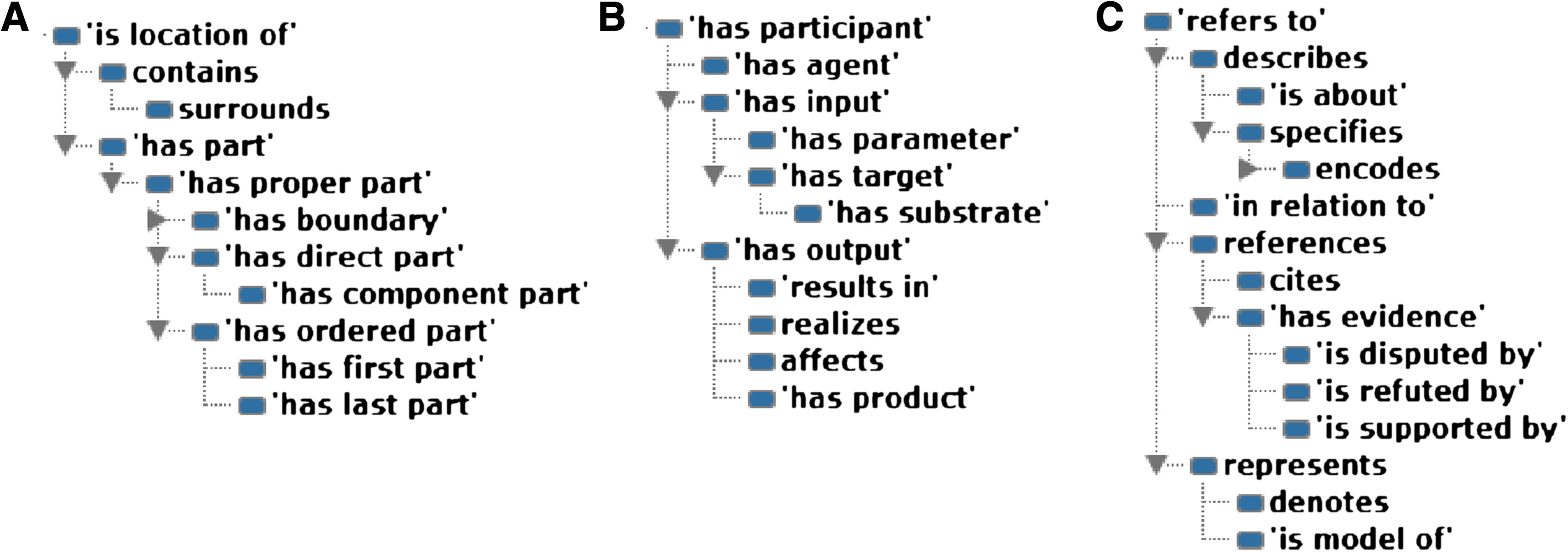

Supplement: Supplementary file 4 — Authors’ original file for figure 3 [file 13326_2013_202_MOESM4_ESM.tiff]

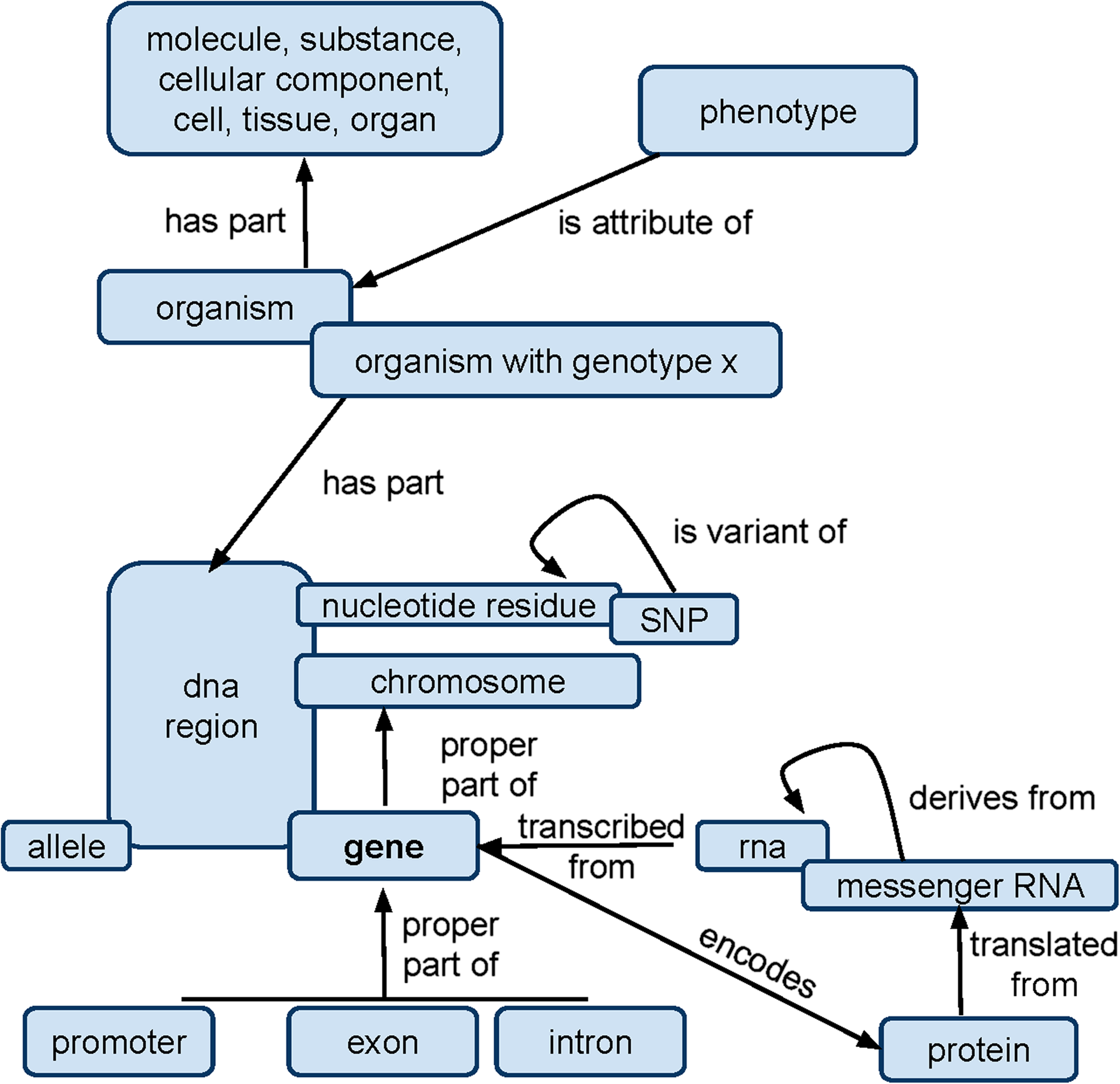

Supplement: Supplementary file 7 — Authors’ original file for figure 6 [file 13326_2013_202_MOESM7_ESM.tiff]

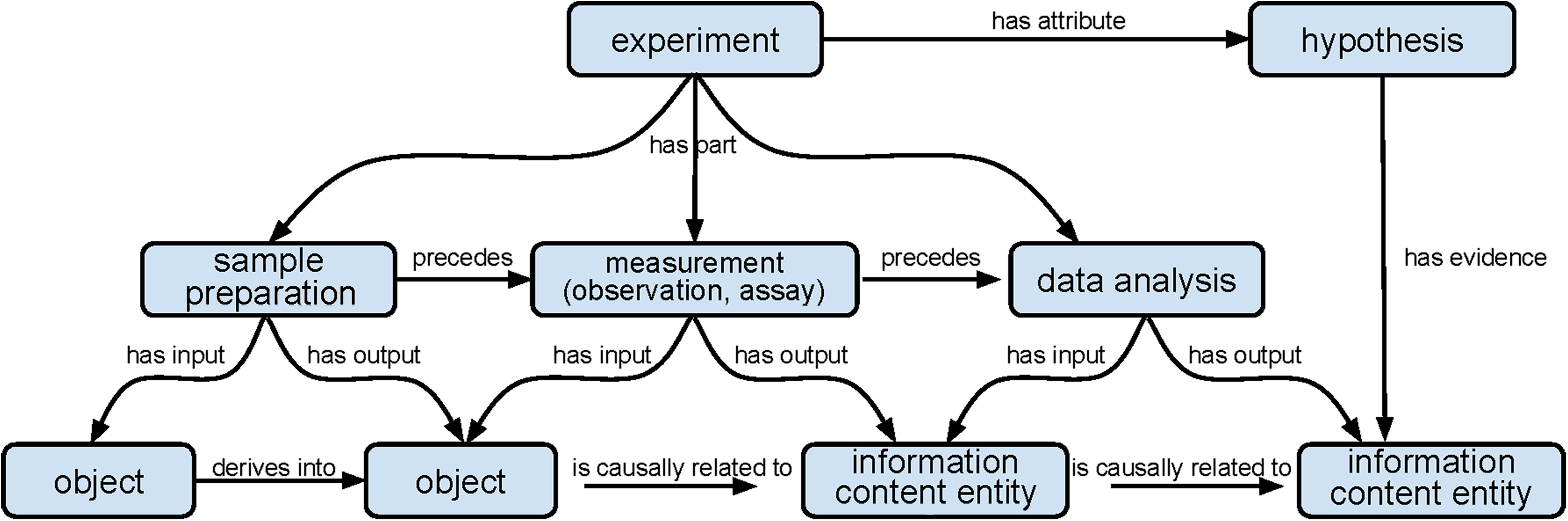

Supplement: Supplementary file 10 — Authors’ original file for figure 9 [file 13326_2013_202_MOESM10_ESM.tiff]
