# Supplementary material for: The Semanticscience Integrated Ontology (SIO) for biomedical research and knowledge discovery
Source: J Biomed Semantics. 2014 Mar 6;5:14. doi: 10.1186/2041-1480-5-14 (PMC4015691; doi:10.1186/2041-1480-5-14)
Supplement: Supplementary file 5 — Authors’ original file for figure 4 [file 13326_2013_202_MOESM5_ESM.pdf]

```
'methane'

equivalentClass
 'molecule'
 and 'has component part' exactly 4 'methane hydrogen atom'
 and 'has component part' exactly 1 'methane carbon atom'
 and 'has component part' only
   ('part of' some 'methane carbon atom' or 'part of' some 'methane hydrogen atom')


'methane hydrogen atom'
equivalentClass
 'hydrogen atom'
 and 'is component part of' exactly 1 'methane'
 and 'is covalently connected to' exactly 1 'methane carbon atom'

'methane carbon atom'
equivalentClass
 'carbon atom'
 and 'is component part of' exactly 1 'methane'
 and 'is covalently connected to' exactly 4 'methane hydrogen atom'
```
