# Supplementary material for: The Semanticscience Integrated Ontology (SIO) for biomedical research and knowledge discovery
Source: J Biomed Semantics. 2014 Mar 6;5:14. doi: 10.1186/2041-1480-5-14 (PMC4015691; doi:10.1186/2041-1480-5-14)
Supplement: Supplementary file 6 — Authors’ original file for figure 5 [file 13326_2013_202_MOESM6_ESM.pdf]

```
'substrate-enzyme phosphorylation by ATP'

equivalentClass
'biochemical reaction'
and 'realizes' some
 ('substrate role' and 'is role of' some 'ATP-substrate enzyme complex')
and 'realizes' some
  ('product role' and 'is role of' some 'ADP-substrate-phosphorylated-enzyme complex')
```
