# Supplementary material for: The Semanticscience Integrated Ontology (SIO) for biomedical research and knowledge discovery
Source: J Biomed Semantics. 2014 Mar 6;5:14. doi: 10.1186/2041-1480-5-14 (PMC4015691; doi:10.1186/2041-1480-5-14)
Supplement: Supplementary file 8 — Authors’ original file for figure 7 [file 13326_2013_202_MOESM8_ESM.pdf]

```
'enzyme-encoding gene'

subClassOf
'gene'
 and 'encodes' some
    ('protein' and 'has function' some 'to covalently modify'
              and 'in relation to' some 'chemical entity'
              and 'is realized in' some 'chemical reaction')
```
