# Supplementary material for: The Semanticscience Integrated Ontology (SIO) for biomedical research and knowledge discovery
Source: J Biomed Semantics. 2014 Mar 6;5:14. doi: 10.1186/2041-1480-5-14 (PMC4015691; doi:10.1186/2041-1480-5-14)
Supplement: Supplementary file 9 — Authors’ original file for figure 8 [file 13326_2013_202_MOESM9_ESM.pdf]

```
'phenotype of an organism with a SNP at position 12'

subClassOf
'phenotype'
 and 'is attribute of' some
      ('organism' that 'has part' some (
       'gene'
        and 'has part' some (
         'snp'
          and 'has value' value 'A'
          and ('has attribute' some (
               'sequence position' that 'has value' value '12')))))
```
