# Supplementary material for: The Semanticscience Integrated Ontology (SIO) for biomedical research and knowledge discovery
Source: J Biomed Semantics. 2014 Mar 6;5:14. doi: 10.1186/2041-1480-5-14 (PMC4015691; doi:10.1186/2041-1480-5-14)
Supplement: Supplementary file 12 — Authors’ original file for figure 11 [file 13326_2013_202_MOESM12_ESM.pdf]

```
SELECT ?go ?label count(distinct ?x)
WHERE {
   {
 # get all the biochemical reactions labelled with protein catabolic process
    ?go rdfs:label ?label .
    FILTER regex(?label, "^protein catabolic process")
    SERVICE <http://biomodels.bio2rdf.org/sparql> {
     ?x <http://semanticscience.org/resource/is-identical-to> ?go .
     ?x a <http://semanticscience.org/resource/biochemical-reaction>
    } # end service
   } UNION {
    # get all the biochemical reactions that are
    # more specific than "protein catabolic process"
    ?go rdfs:label ?label .
 ?go rdfs:subClassOf+ ?tgo . # get all the subclasses of the target to term
    ?tgo rdfs:label ?tlabel .
    FILTER regex(?tlabel, "^protein catabolic process")
    SERVICE <http://biomodels.bio2rdf.org/sparql> {
     ?x < http://semanticscience.org/resource/is-identical-to> ?go .
     ?x a < http://semanticscience.org/resource/biochemical-reaction>
    } # end service
   } # end union
} # end where
```
