# Supplementary material for: The Semanticscience Integrated Ontology (SIO) for biomedical research and knowledge discovery
Source: J Biomed Semantics. 2014 Mar 6;5:14. doi: 10.1186/2041-1480-5-14 (PMC4015691; doi:10.1186/2041-1480-5-14)
Supplement: Supplementary file 13 — Authors’ original file for figure 12 [file 13326_2013_202_MOESM13_ESM.pdf]

```
PREFIX sio: <http://semanticscience.org/resource/>
PREFIX kegg: <http://lsrn.org/KEGG_PATHWAY:>
SELECT ?participant ?protein ?chemical
WHERE {
kegg:hsa00232 sio:SIO_000132 ?gene .# caffeine_metabolism has-participant?gene

 ?gene sio:SIO_010078 ?protein .     # ?gene encodes ?protein
}
```
