# Supplementary material for: The Semanticscience Integrated Ontology (SIO) for biomedical research and knowledge discovery
Source: J Biomed Semantics. 2014 Mar 6;5:14. doi: 10.1186/2041-1480-5-14 (PMC4015691; doi:10.1186/2041-1480-5-14)
Supplement: Supplementary file 14 — Authors’ original file for figure 13 [file 13326_2013_202_MOESM14_ESM.pdf]

```
@prefix nanopub: <http://www.nanopub.org/nschema#> .
@prefix rdfs: <http://www.w3.org/2000/01/rdf-schema#> .
@prefix sio: <http://semanticscience.org/resource/> .
@prefix xsd: <http://www.w3.org/2001/XMLSchema#> .
@prefix :
<http://rdf.biosemantics.org/vocabularies/gene_disease_nanopub_example#> .

:NanoPub_1_Assertion {
 :Association_1
  a sio:gene-disease-association ;
  sio:has-measurement-value :Association_1_p_value ;
  sio:refers-to <http://bio2rdf.org/ncbigene:55835>,
<http://bio2rdf.org/omim:210600> ;
  rdfs:comment "This association has p-value of 0.00066, has attribute gene
CENPJ (NCBI gene id 55835) and attribute disease Seckel Syndrome (OMIM
210600)."@en .

 :Association_1_p_value
  a sio:probability-value ;
  sio:has-value "0.000656211037469712"^^xsd:float .
}
```
