# Supplementary material for: The Semanticscience Integrated Ontology (SIO) for biomedical research and knowledge discovery
Source: J Biomed Semantics. 2014 Mar 6;5:14. doi: 10.1186/2041-1480-5-14 (PMC4015691; doi:10.1186/2041-1480-5-14)
Supplement: Supplementary file 11 — Authors’ original file for figure 10 [file 13326_2013_202_MOESM11_ESM.pdf]

```
sio:    <http://semanticscience.org/resource/>
obo:    <http://purl.obolibrary.org/obo/>
taxon:  <http://bio2rdf.org/taxon:>

:p rdfs:label "Rob" .                   # Rob
:p a taxon:9606 .                       # Rob is human, as per the NCBI taxonomy in
Bio2RDF
:p sio:has-attribute :m1 .              # Rob has an attribute m1

:m1 rdfs:label "Rob's mass on Jan 15, 2012"@en      # Rob's mass
:m1 a sio:mass .                                    # m1 is a mass
:m1 sio:has-value "74.5"^^xsd:float .               # the value is 74.5
:m1 sio:has-unit obo:UO_0000009 .                   # the unit is kilogram as
                                                    #   per the Unit Ontology
:m1 sio:measured-at :t1 .                           # mass measured at t1

:t1 a sio:time-instant .
:t1 sio:has-value "2012-01-15T12:03:00-05:00"^^xsd:dateTime .

:mp a sio:measuring .                   # measuring process
:mp sio:has-agent  :robscale .          # measuring involved rob's scale
:mp sio:has-output :m1 .                # measuring resulted in a measurement value

:robscale rdfs:label "Rob's scale"@en  # Rob's scale
:robscale a obo:MMO_0000217 .          # body measurement scale as per the Measurement
                                       #  Method Ontology
```
